# Supplementary material for: Privatization of Biofilm Matrix in Structurally Heterogeneous Biofilms
Source: mSystems. 2020 Aug 4;5(4):e00425-20. doi: 10.1128/mSystems.00425-20 (PMC7406226; doi:10.1128/mSystems.00425-20)
Supplement: FIG S1 [file mSystems.00425-20-sf001.pdf]

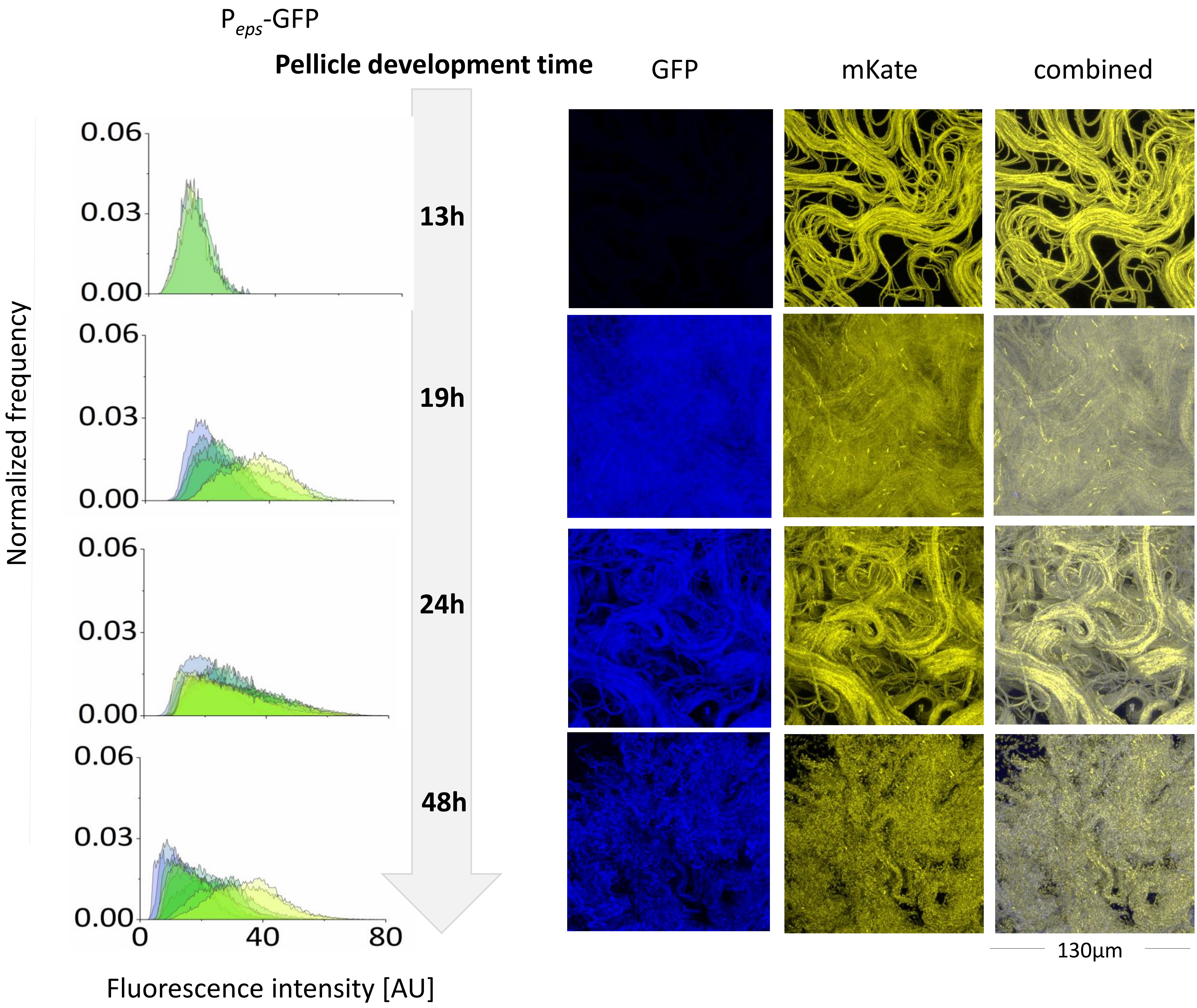
P$_{eps}$-GFP
Pellicle development time
GFP
mKate
combined
Normalized frequency
0.06
0.03
0.00
13h
19h
24h
48h
0
40
80
Fluorescence intensity [AU]
130µm
