# Supplementary material for: Privatization of Biofilm Matrix in Structurally Heterogeneous Biofilms
Source: mSystems. 2020 Aug 4;5(4):e00425-20. doi: 10.1128/mSystems.00425-20 (PMC7406226; doi:10.1128/mSystems.00425-20)
Supplement: FIG S2 [file mSystems.00425-20-sf002.pdf]

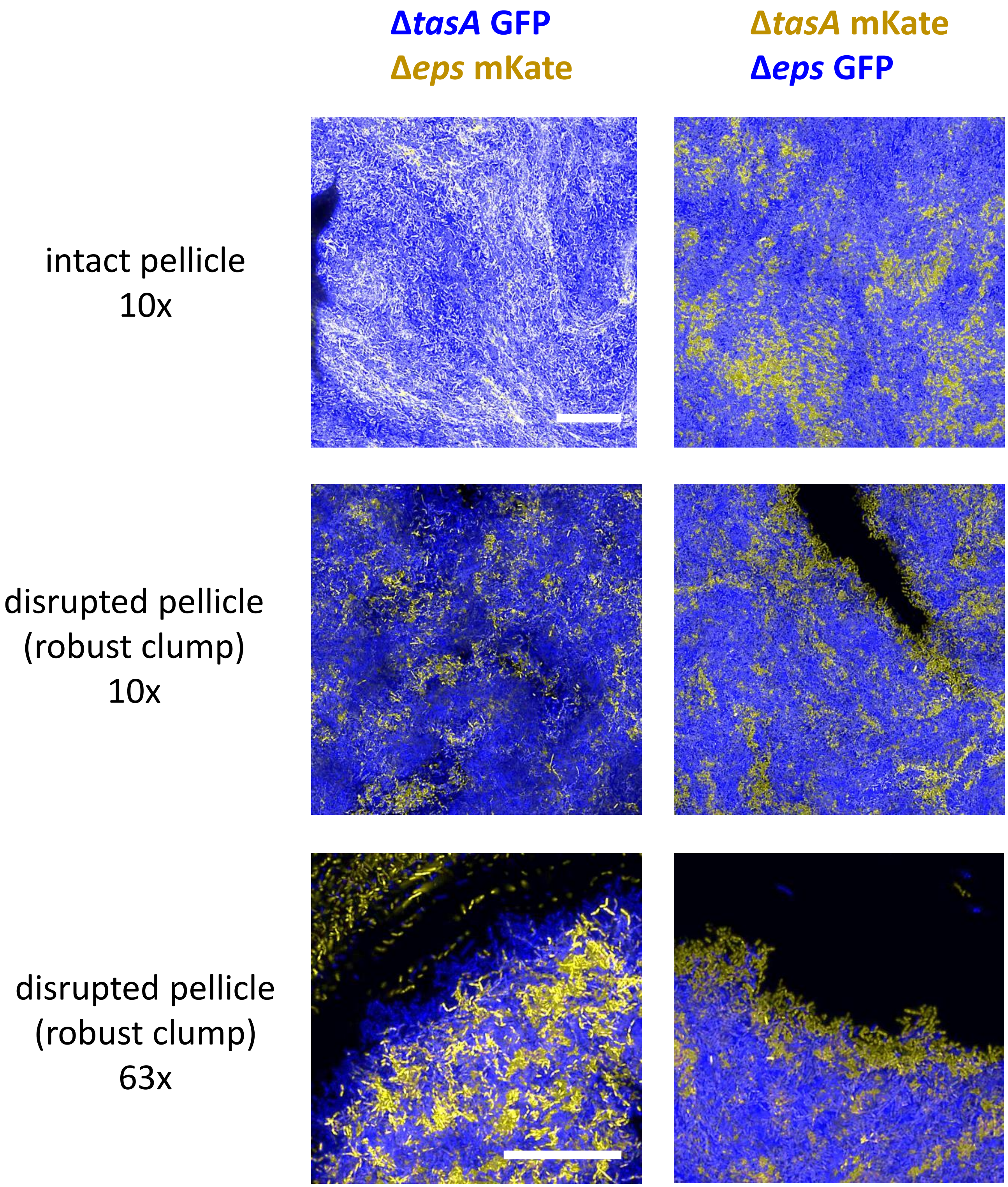

ΔtasA GFP
Δeps mKate
ΔtasA mKate
Δeps GFP
intact pellicle
10x
disrupted pellicle
(robust clump)
10x
disrupted pellicle
(robust clump)
63x
