# Supplementary figures and images for: Privatization of Biofilm Matrix in Structurally Heterogeneous Biofilms
Source: mSystems. 2020 Aug 4;5(4):e00425-20. doi: 10.1128/mSystems.00425-20 (PMC7406226; doi:10.1128/mSystems.00425-20)

WT

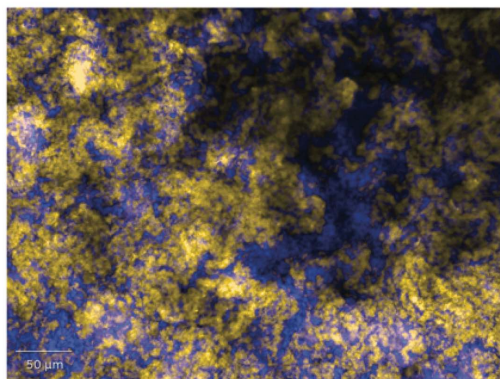

$\Delta eps$

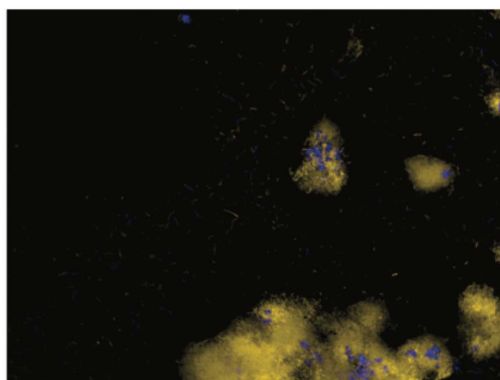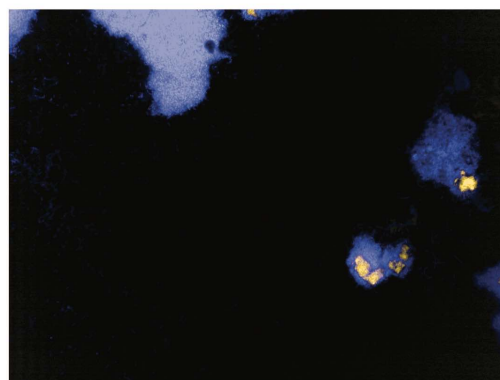

$\Delta tasA$

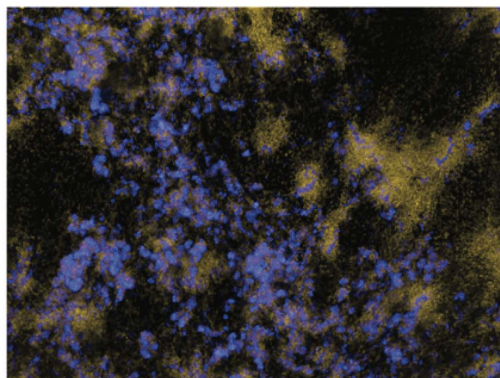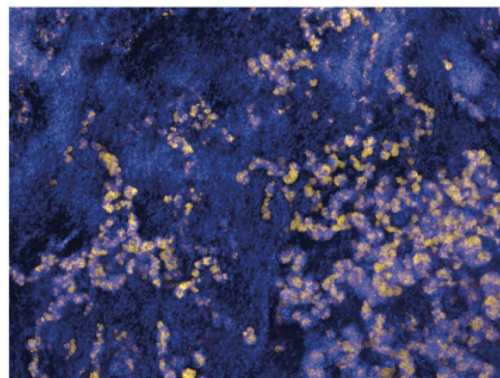

$\Delta eps \Delta tasA$

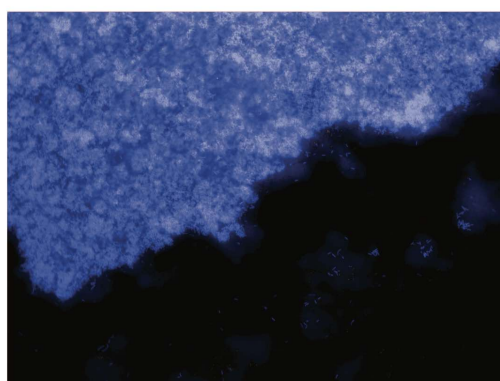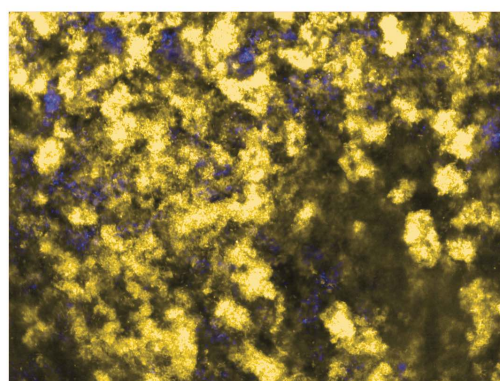

WT GFP  
mutant mKATE

WT mKATE  
mutant GFP

Supplement: FIG S3 [file mSystems.00425-20-sf003.pdf]

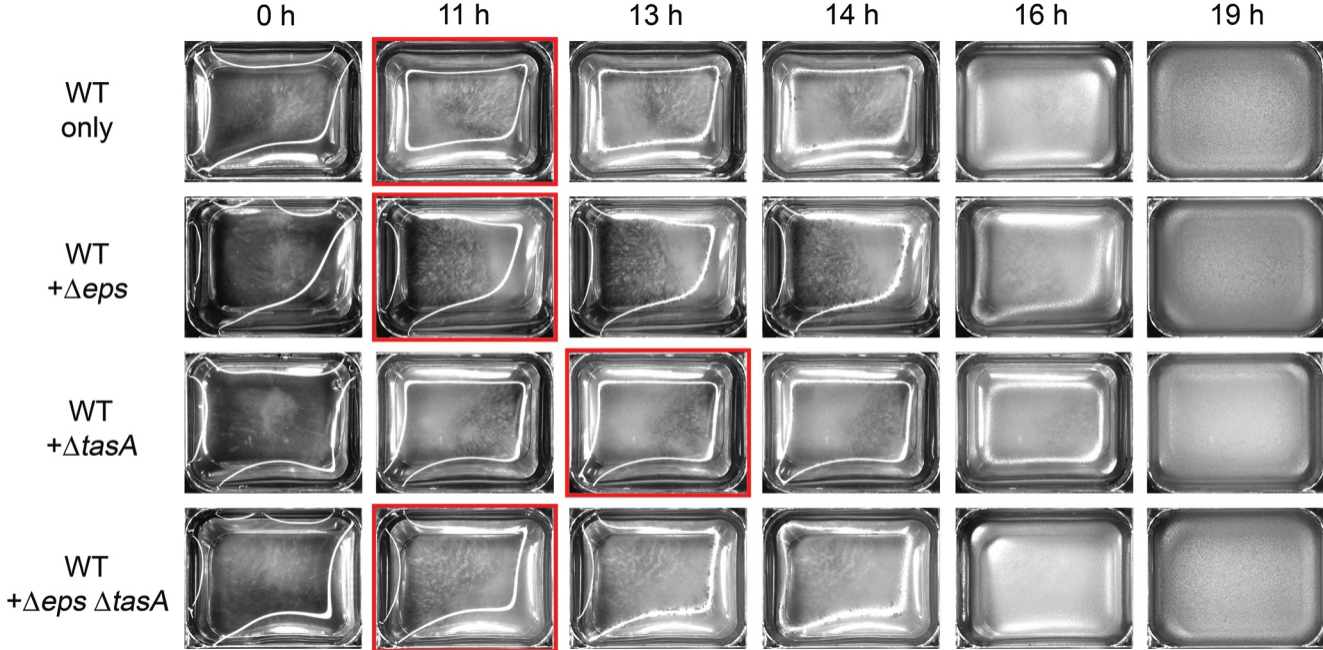

Supplement: FIG S4 [file mSystems.00425-20-sf004.pdf]
